# *Gentiana asclepiadea* L. from Two High Mountainous Habitats: Inter- and Intrapopulation Variability Based on Species’ Phytochemistry

**DOI:** 10.3390/plants10010140

**Published:** 2021-01-12

**Authors:** Zorica Popović, Dijana Krstić-Milošević, Milena Marković, Vera Vidaković, Srđan Bojović

**Affiliations:** 1 Department of Ecology, Institute for Biological Research “Siniša Stanković”—National Institute of Republic of Serbia, University of Belgrade, Bulevar despota Stefana 142, 11000 Belgrade, Serbia; milenastefanovic@ibiss.bg.ac.rs (M.M.); vera.vidakovic@ibiss.bg.ac.rs (V.V.); bojovic@ibiss.bg.ac.rs (S.B.); 2 Department of Plant Physiology, Institute for Biological Research “Siniša Stanković”—National Institute of Republic of Serbia, University of Belgrade, Bulevar despota Stefana 142, 11000 Belgrade, Serbia; dijana@ibiss.bg.ac.rs

**Keywords:** willow gentian, geographically distinct populations, secondary metabolites, inter-and intrapopulation variability

## Abstract

Natural populations of *Gentiana asclepiadea* L., located at two mountainous sites, were HPLC-analyzed regarding the contents of six representative secondary metabolites. The contents of swertiamarin (SWM), gentiopicrin (GP), sweroside (SWZ), mangiferin (MGF), isoorientin (ISOOR), and isovitexin (ISOV) were determined in six populations (three per study site), and separately for aboveground and belowground plant parts. PCA showed a clear separation of four groups according to the contents of the analyzed secondary metabolites. Out of six analyzed compounds, five were present in all samples and only one (SWZ) was found in Golija populations (belowground parts) but not in Vlasina populations, and its presence can be indicative of the geolocation of populations. Clear separation of groups was mostly affected by the different contents of chemical compounds in plant parts (aboveground versus belowground) and by the differences related to population origin (higher content of SWM and GP in belowground parts of individuals from Vlasina populations and higher content of MGF and ISOOR of individuals from Golija populations). The results of this study contribute to the spatiochemical profiling of *G. asclepiadea* populations and a better understanding of inter- and intrapopulation variability of pharmacologically important compounds.

## 1. Introduction

The genus *Gentiana* comprises about 400 species primarily distributed throughout the Eurasian mountainous regions, while some species are found in lowlands and some are distributed in diverse habitats in the Andes, India, New Zealand, and Southern Australia [1]. Most gentian species are valued as medicinal and ornamental plants and are intensively researched due to the presence of pharmacologically important phytochemicals. Gentians are prescribed by many traditional pharmacopoeias and incorporated into more than a hundred official drugs, owing to their most important active constituents, secoiridoid glycosides and xanthones [2].

*Gentiana asclepiadea* L., Willow gentian, native to mountains of Central and Southern Europe, is 1 of 11 gentian species present in Serbian flora [3]. It mostly occurs at high altitudes approaching the subalpine belt, at mountain ores, wet meadows, peat bogs, light forests, and forest edges, and is most frequently found in spruce forests. Perennial species (15–60 cm high) blooming from July to September are characterized by the strong, thick rhizome that is used as a traditional remedy for hepatitis infections and digestive problems [4].

High-altitude (over 1000 m) medicinal and aromatic flora deserve special attention due to their richness in valuable secondary metabolites, which become more abundant with increasing elevation [5]. The distribution of herbaceous mountainous flora is determined by specific topography, mountains’ directional extensions, slope, soil type, vegetation cover, and so forth. [6]. The high mountainous habitat is characterized by a set of environmental conditions, namely, temperature (including day–night temperature ranges), precipitation, insolation and UV radiation, duration of vegetation season, day length, and clear sky conditions, which together contribute to plants’ adaptive response regarding both primary and secondary metabolism [7]. Among the numerous environmental influences that have a synergistic impact on plant growth and reproduction, UV-B radiation is considered to be the most important factor for photosynthetic production and secondary metabolism. Under field conditions, plant biomass accumulation and phenophase shifting occur continually throughout the whole growing season, despite the negative effect of UV-B radiation on the photosynthetic apparatus [8]. Nevertheless, a substantial amount of energy produced in photosynthesis is required to acclimate and restore the damage caused by UV-B to photosynthetic pigments and cellular metabolism [9]. Light environment adaptation (UV-B radiation adaptation) at the secondary metabolism level is indicated by the enhancement of biosynthesis and aggregation of protecting UV-B compounds, namely, flavonoids (with strong free-radical scavenging potential) and sinapate esters (capable of absorption in the wavelength range from 280 to 340 nm) [10,11,12,13]. 

Other important external drivers for biosynthesis and accumulation of secondary metabolites are the attraction of pollinators, which decrease both in diversity and activity in high altitudes [14], and intensive herbivore pressure, considering that plant-eating insects have limited food choice to complete their life cycle [15]. High levels of biologically active secondary metabolites (potentially poisonous) in high-altitude habitats also contribute to shaping the community structure, as certain plant species are avoided by grazers. Low palatability (and in higher amounts toxicity) of gentians is an important grazing indicator and the cause for their increased abundance from the Neolithic period, as revealed by pollen records [16].

Eco-biochemical studies of natural populations are important for understanding species’ responses to specific site conditions in high-altitude environments, while plant-part-related differences provide further information on specific environmental drivers that are predicted to cause ecophysiological and metabolic adaptations. This research aimed to assess geographic variability, interpopulation variability, and plant-part-related differences in natural populations of Willow gentian based on the contents of six representative secondary metabolites.

## 2. Results

**Figure 1:**
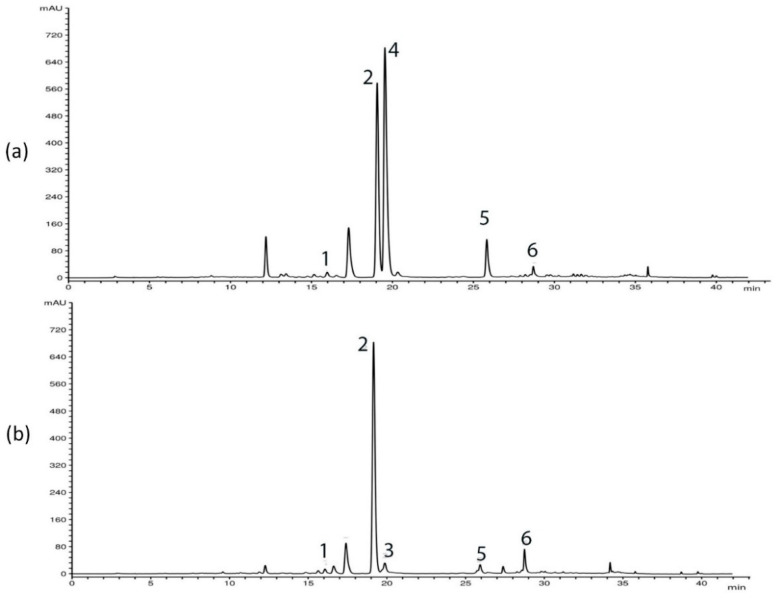
HPLC profiles (λ = 260 nm) of *Gentiana asclepiadea* methanol extracts of plants collected from Golija: (**a**) aboveground parts; (**b**) belowground parts. Peaks: swertiamarin (1), gentiopicrin (2), sweroside (3), mangiferin (4), isoorientin (5), and isovitexin (6).

**Figure 2:**
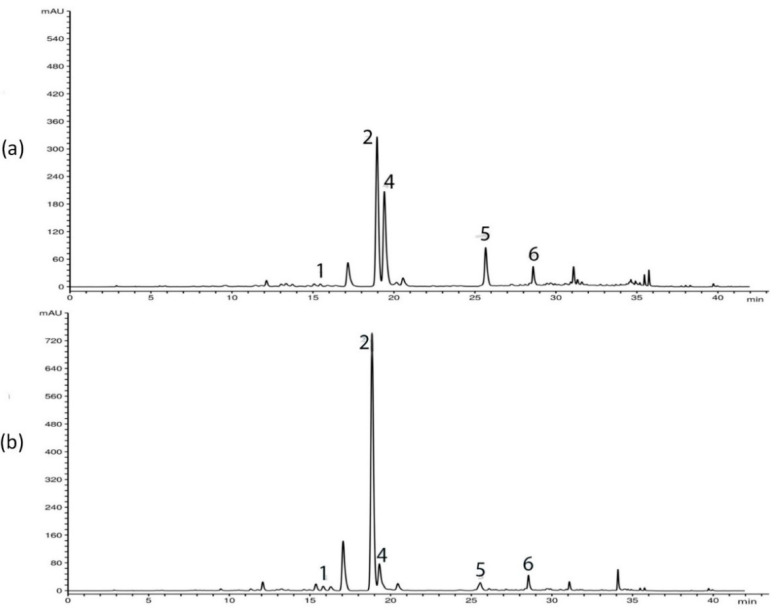
HPLC profiles (λ = 260 nm) of *Gentiana asclepiadea* methanol extracts of plants collected from Vlasina: (**a**) aboveground parts; (**b**) belowground parts. Peaks: swertiamarin (1), gentiopicrin (2), mangiferin (4), isoorientin (5), and isovitexin (6).

**Figure 3:**
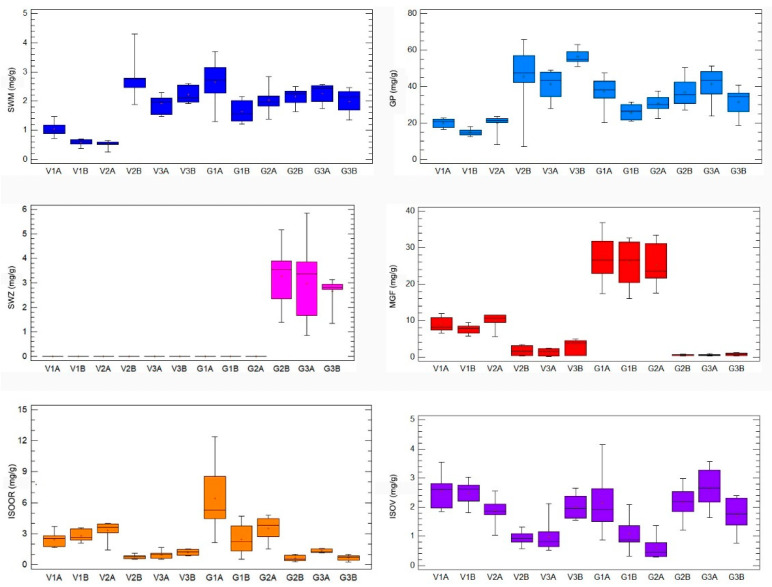
The contents of six representative secondary metabolites (mg/g dry weight (dw)) in *Gentiana asclepiadea* by 12 groups of samples (original, nontransformed data): Vlasina aboveground plant parts (V1A, V2A, V3A), Vlasina belowground plant parts (V1B, V2B, V3B), Golija aboveground plant parts (G1A, G2A, G3A), Golija belowground plant parts (G1B, G2B, G3B). Boxplot major features (bottom to top): minimum value, box (interquartile range), and maximum value. Horizontal bar inside a box is median. Cross inside a box is mean value. Vertical bar is range.

HPLC analysis of *G. asclepiadea* plants revealed the presence of six major compounds—secoiridoid glucosides swertiamarin (SWM), gentiopicrin (GP), and sweroside (SWZ); xanthone mangiferin (MGF); and two *C*-glucoflavones isoorientin (ISOOR) and isovitexin (ISOV). Aboveground and belowground plant parts contained varying contents of these compounds. Gentiopicrin was detected as the most abundant compound in all analyzed samples. Chromatograms of above- and belowground plant parts with identified peaks of secondary metabolites are presented in Figure 1 (Golija) and Figure 2 (Vlasina). The contents of six analyzed secondary metabolites (mg/g dry weight (dw)) in above- and belowground plant parts from Vlasina and Golija populations are shown in Figure 3.

The impacts of three factors (location, population, and plant part) on dependent variables (contents of secondary metabolites) are presented in Table 1. The applied model for nested ANOVA is highly significant for each analyzed property (*p* < 0.001; not shown). It is noticeable that for each property, there are statistically significant differences only between plant parts (above- and belowground). For half of the examined properties, there are differences between localities (Golija and Vlasina), while none of the properties show statistically significant differences between populations.

**Figure 4:**
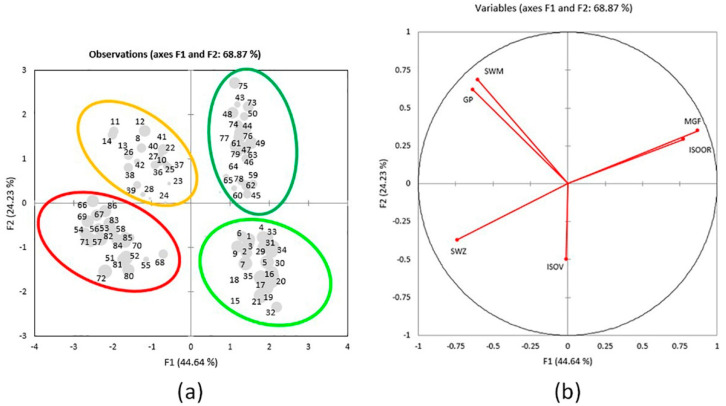
(**a**) Score plot. Separation of 86 individuals in regard to interactions of parts of a plant and locality according to the contents of 6 variables in the plane of the first 2 axes. Larger-dot elements are better-represented elements, with the larger square cosine of the observations for the first two axes: 1–7 = V1A; 8–14 = V1B; 15–21 = V2A; 22–28 = V2B; 29–35 = V3A; 36–42 = V3B; 43–50 = G1A; 51–57 = G1B; 58–64 = G2A; 65–72 = G2B; 73–79 = G3A; 80–86 = G3B; (**b**) Loading plot. Structure of the six variables in the plane of the first 2 axes in 86 individuals.

PCA separated 86 elements into 4 distinct assemblages (Figure 4a). The results show that the first two axes explain 68.87% of data variability (44.64% and 24.23%, respectively), and the information about well-compressed data was confirmed by their eigenvalues > 1. Based on the sum of squared correlations between the variables and factors in the PC 1–2 plane (sum of r^2^ > 0.87), it can be concluded that MGF, SWM, and GP are the best-represented properties and are likely to have the most important role in the total variability of the sample (Table 2). The formation of the first axis is mainly defined by the variability of MGF, ISOOR, SWZ, and GP, whereas the variability of SWM is the most important for the formation of the second axis. The structure of the properties and their relationships are presented in Figure 4b. The tendencies among the elements (i.e., their groupings (or separation)) are noticeable in the plane of the first two axes (Figure 4a). Well-represented elements, with the larger square cosine of the observations for the first two axes, are displayed by larger dots.

The separation of 86 elements by different plant parts is clear, as the aboveground plant parts are grouped on the right part of the figure (Golija top right and Vlasina bottom right) and the belowground plant parts are grouped on the left part (Vlasina top left and Golija bottom left) (Figure 4a). The red ellipse encompasses elements that contain SWZ, and these are belowground parts from populations G1, G2, and G3 (G1B, G2B, and G3B). The yellow ellipse encompasses elements with the higher content of SWM and GP and these are belowground part populations V1, V2, and V3 (V1B, V2B, and V3B) (the only exception is element nine, which is poorly represented in the plane of the first two axes). A dark-green ellipse encompasses elements with the higher content of MGF and ISOOR and these are aboveground parts from populations G1, G2, and G3 (G1A, G2A, and G3A). A light-green ellipse encompasses elements with the lower content of SWM and GP and these are aboveground parts from populations V1, V2, and V3 (V1A, V2A, and V3A).

## 3. Discussion

Information on the chemical composition of essential oils and extracts obtained from *G. asclepiadea* has been provided in numerous publications, mostly aimed at evaluation of their biological activities [17]. The volatile profile of flowers and leaves was reported for the natural population of willow gentian [18], and Kozuharova et al. [19] suggested that specific secondary metabolites may have an important role against seed predators. However, information about locality-dependent differences and inter- and intrapopulation variability based on species’ phytochemistry has not been reported to date.

Five of six analyzed secondary metabolites were detected in all samples of *G. asclepiadea*, and only one (SWZ) which was found in Golija populations but not in Vlasina populations can be considered as a distinctive compound between the two geographic locations. However, this finding should be interpreted with a note that it relates to only one sampling period, and the occurrence of SWZ in earlier developmental stages of the Vlasina population should not be excluded. Although high ontogenetic variability in the content of secondary metabolites has been documented, the same gender-specific compounds were present in the preflowering, flowering, and postflowering stages in *Gentiana pneumonanthe* [20]. Considering that the full maturity phase at the time of flowering is the period of collection and harvesting of medicinal plants [21], the information regarding the Vlasina populations is important and deserves further research. Gentiopicrin was the most abundant compound in all samples (36.36 ± 7.53 mg/g and 26.98 ± 5.04 mg/g in aboveground parts of Golija and Vlasina populations, respectively; 31.28 ± 6.56 mg/g and 38.96 ± 8.22 mg/g in belowground parts of Golija and Vlasina populations, respectively). It was also a dominant component in belowground parts of the natural populations of *G. asclepiadea* from Hungary (varying from 45 to 68 mg/g depending on the sampling site), and SWM and SWZ were also found in all studied populations [22]. Gentiopicroside was the dominant iridoid in Caucasian populations of *G. asclepiadea* (about 90 mg/g in herbs and 65 mg/g in roots, respectively), SWM was detected at a lower level (about 1.5 mg/g in herbs and 6 mg/g in roots, respectively), and SWZ was only found in trace amounts in all plant parts [23]. The content of MGF in the aboveground plant parts found in the populations from Golija (17.62 ± 4.03 mg/g) was very similar to that previously reported for Caucasian populations (17.48 ± 0.33 mg/g), although it was lower in the populations from Vlasina (6.49 ± 1.04 mg/g). However, this xanthone compound was detected in belowground parts of the populations from Vlasina and Golija but not in the populations from Azerbaijan [23]. Flavonoids ISOOR and ISOV, which are predominantly abundant in aboveground plant parts of gentians, were detected in all plant parts of *G. asclepiadea*, comparable to a previous report [23].

Differences between individuals from different locations are also confirmed by the higher content of SWM and GP in belowground parts obtained from Vlasina individuals and the higher content of MGF and ISOOR in aboveground parts of Golija individuals. Besides specific local climatic and topoedaphic conditions at two localities, sampled populations were members of different forest stands (beech-dominated in Vlasina and spruce-dominated in Golija), and differences in local flora and the composition of plant communities may also affect the production of secondary metabolites. Comparative analyses related to phytochemical profiling of geographically distinct populations of numerous species revealed the need for more research regarding the impact of both genetic origin and a specific set of environmental conditions on the composition and content of secondary metabolites [24]. Several studies documented the chemical variability among natural populations in *Gentiana* species [20,25,26,27,28,29,30]. The study of high-altitude *G. straminea* showed significant interpopulation variability for several secondary metabolites, providing the basis for a geo-authentic production area for this traditional medicinal plant [28], and our results show that particular metabolites can even be absent in the chemical profile of some populations. Sweroside, a secoiridoid with anti-inflammatory and analgesic properties, has been used in the treatment against osteoporosis in traditional Chinese medicine and can be a promising therapeutic product for this medical issue [31]. Information regarding its abundance in certain geolocations can be valuable for further investigation regarding the plant–environment biochemical response and indicative of exploitation and production.

The most prominent differences in the presence and content of the six secondary metabolites were recorded between distinct plant parts, which is in accordance with the rich literature data on the distribution of main chemical compounds in gentians [2]. Secoiridoids are predominantly present in the belowground plant parts but can also be found in aboveground parts at a prominent level [32]. Isoorientin and isovitexin are the most frequently found *C*-glucoflavones in *Gentiana* sp., confirmed in all representatives of a genus [33,34]. Flavonoids are a large group of phenolic compounds with pronounced antioxidant and chelating effects, and their ultraviolet-absorbing properties are an important adaptive feature at higher altitudes [35]. The accumulation of flavonoids occurs in the preflowering period and then reaches the maximum value during flowering [36]. Iridoids and secoiridoid glycosides have a primarily defensive function against herbivores and pathogens and can be detected in different plant organs [32]. The high amount of these compounds in belowground organs, which is found in all ontogenetic stages for gentians [20,37], may imply a response to specific biotic pressures. However, more information regarding species autecology (i.e., photosynthesis, morphology, biotic interactions) would provide a better understanding of plant–environment relations among gentians [38,39]. 

For a large number of phytochemically investigated plants, specific secondary metabolites have been determined exclusively or predominantly in a particular organ, and most of the differences in the quantitative and/or qualitative composition of secondary metabolites are reported between the below- and aboveground plant parts [40]. Different chemical compositions of below- and aboveground plant parts arise from the different environmental pressures to which they are exposed. The secondary metabolites predominantly present in aboveground plant organs have multiple functions, as pollinator attractants (volatile attractants in flower petals) [41], for seed dispersal (esters in fruits) [42], toxic and repellent functions against herbivores and pathogens (phenolic glucosides, furanocoumarins, tannins in aerial parts, alkaloids in nectar) [43], and in protection against abiotic factors (flavonoids in epidermal tissues for UV protection) [35]. In belowground parts of the plant, the most important function of secondary metabolites (e.g., emodin) is a defense against herbivores that can seriously damage or destroy the root and consequently the whole plant [44], but their allelopathic and self-regulating functions are achieved as well (phenolic acids, flavanols, flavones, flavanones, anthocyanins) [45]. Regarding the pharmacological activities of a plant species, the “medicinal part” of a plant is usually indicated either in pharmacognostic literature or ethnopharmacobotanical sources [21].

Roots are recognized to be able to synthesize and accumulate a diversity of secondary metabolites in response to environmental stressors [46], and significant variability between the root and shoot defensive chemicals may be caused by habitat conditions [47,48]. In reviewing the root/shoot differences regarding the secondary metabolites, Rasmann and Agrawal [49] pointed that (1) compounds present in aboveground parts are most often present in roots; (2) considering their surface area and exposition to belowground herbivores, roots are pronouncedly chemically defended; and (3) even though differential allocation to aboveground and belowground parts is not completely clarified, it depends on the plant family, species, genotype, and ontogenetic stage of tissues.

## 4. Materials and Methods

### 4.1. Study Sites

The Vlasina Plateau is located in southeastern Serbia (42°43′55″ N, 22°19′18″ E) at an altitude of over 1200 m, surrounded by the mountains of Rhodope massif: Bukova glava, Čemernik, Plana, and Vardenik. Mountains around the plateau are very rich in springs that form streams and smaller rivers, and along with watercourses from the plateau, they flow into the Vlasina Lake located in the center of the plateau. Edaphic factors are determined by topography (plateau and surrounding mountains) and soils (peat and district brown forest soils). The climate is humid and submountainous, with cool summers and very cold winters. Lake Vlasina and the surrounding area are classified in the first protection category—Outstanding Natural Landscape “Vlasina”—due to the exceptional richness of flora and fauna, both freshwater and terrestrial (under state protection since 2006). The vegetation of the area is a unique mosaic of meadows, pastures, and high-altitude forests (birch, beech, pine, and juniper).

Golija Mountain is part of the Dinaric mountain range, situated in southwestern Serbia (43°20′16″ N, 20°16′36″ E), with the highest peak Jankov kamen at 1833 m. Climatic patterns in this area are moderate continental; in higher zones (over 1300 m), the climate is mountainous with severe winters with high snow cover and short summers. Overall edaphic, hydrological, and climatic conditions and the refugial character of many habitats lead to a great diversity of flora and fauna, which is the reason for a high degree of protection within the Golija–Studenica Biosphere Reserve, the first UNESCO-MAB registered biosphere reserve in Serbia (2001). Golija is the most forested Serbian mountain, with the largest, best-preserved, and highest-quality forest complexes, with primeval forest patches. Deciduous and deciduous–coniferous old-grown forests (beech, oak, beech–fir, and beech–spruce) predominate along the elevational gradient, and above 1700 m, only spruce is represented.

**Figure 5:**
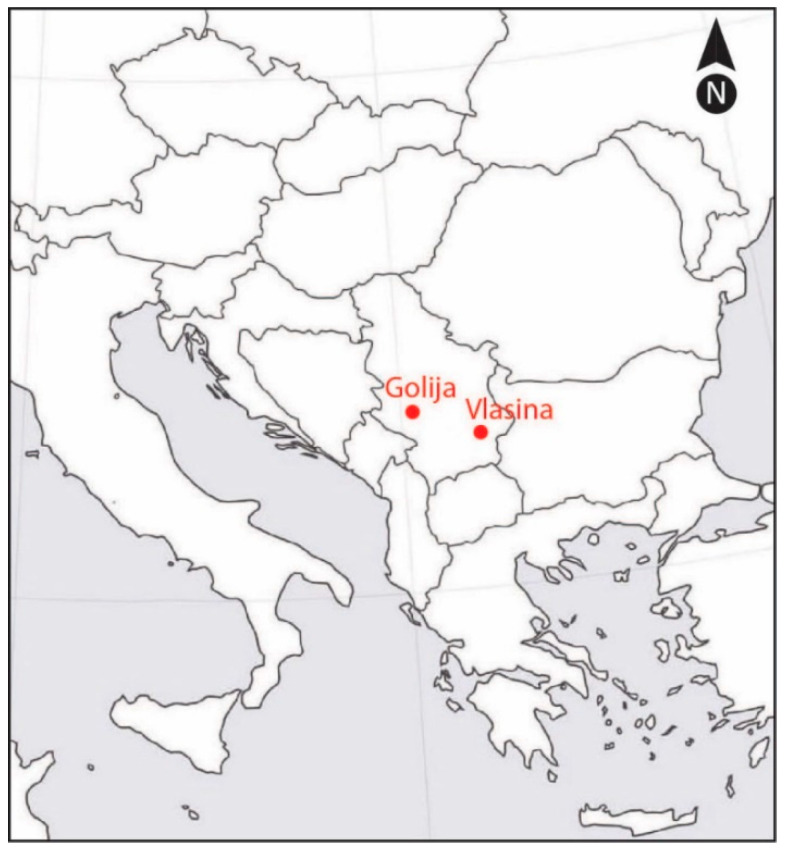
The positions of the Vlasina and Golija study sites.

The positions of the Vlasina and Golija study sites are shown in Figure 5. Geographical coordinates of the studied populations and climate characteristics of locations are given in Table 3.

### 4.2. Plant Material and Sample Preparation

At both study sites—Vlasina (V) and Golija (G)—individuals from three distinct populations of *G. asclepiadea* were sampled during the flowering period (August 2019). All specimens were healthy individuals without any visible damage caused by herbivores or pathogens. Populations were positioned at the edges of forest communities dominated by beech, beech–birch (Vlasina), or spruce and beech–spruce (Golija), with the distance between them of about several kilometers. From one population, seven to eight individuals were sampled; from each individual, a mixed sample of three stems was considered as the aboveground part (A), and the common rhizome as the belowground part (B). Above- and belowground parts were divided after air-drying of whole plants and analyzed separately. The total number of samples was 2 (locations) × 3 (populations) × 7–8 (individuals) × 2 (plant parts) = 86. Representative samples are in the herbarium at the Institute for Biological Research, Belgrade (voucher codes V1, V2, V3, G1, G2, G3). 

### 4.3. Chemical Analysis

#### 4.3.1. Reagents and Chemicals

Standard compounds mangiferin, isoorientin, and isovitexin were purchased from Sigma-Aldrich (Steinheim, Germany). Swertiamarin, gentiopicrin, and sweroside were purchased from Cfm Oscar Tropitzsch (Marktredwitz, Germany). 

#### 4.3.2. Extraction

Each sample of *G. asclepiadea* was divided into aboveground and belowground plant parts (A—stems, leaves, and reproductive parts, B—roots and rhizomes) and analyzed separately. Air-dried samples were ground to a fine powder (250 mg) and extracted with 5 mL of methanol in an ultrasonic bath for 20 min. After sonication, extraction was continued by maceration for 48 h in the dark at room temperature. The extracts were filtered into 5 mL volumetric flasks, adjusted to the volume with methanol, and stored at 4 °C until use for HPLC analysis.

#### 4.3.3. HPLC Analysis

Chromatographic analysis of the secondary metabolites was carried out on an Agilent series 1100 HPLC instrument (Agilent Technologies, Waldronn, Germany) with a DAD, on a reverse-phase Zorbax SB C-18 (Agilent, Newport, Delaware, USA) analytical column (250 × 4.6 mm, 5 µm particle size) thermostatted at 25 °C. Prior to HPLC analysis, the extracts were filtered through nylon syringe filters (Captiva syringe filters, 0.45 µm, 13 mm, Agilent Technologies). The mobile phase consisted of solvent A (1%, *v*/*v* solution of orthophosphoric acid in water) and solvent B (acetonitrile, J.T. Baker, Deventer, the Netherlands), using gradient elution previously published by Popović et al. [20]. Briefly, samples of belowground and aboveground plant parts were separated as follows: 98–90% A 0–5 min, 90–85% A 5–17 min, 85% A 17–20 min, 85–70% A 20–30 min, 70–0% A 30–39 min, and 0% A 39–42 min. The flow rate was 1 mL/min. The injection volume of samples was 5 µL; the detection wavelengths were set at 260 and 320 nm. The quantification of secondary metabolites was done using the external standard method by preparing calibration standards ranging from 0.01 to 0.5 mg/mL and recording the calibration curves at 260 nm for secoiridoids and flavonoids, and at 320 nm for xanthone mangiferin. The results are presented as milligrams per gram of dry weight (dw).

### 4.4. Statistical Analysis

A total of 516 numerical data points related to *G. asclepiadea* were analyzed: two mountainous sites (V—Vlasina and G—Golija) × three populations (V1, V2, V3; G1, G2, G3) × two plant parts (A—aboveground and B—belowground) × seven (eight) elements (from seven to eight plants per population) × six secondary metabolites (SWM, GP, MGF, SWZ, ISOOR, and ISOV). The choice of statistical tests was made based on preliminary verification of conditions for the application of the majority of the parametric tests: normal distribution (chi-square test, *p* ≥ 0.05) and the equality of variances (Levene’s and Batlett’s tests, *p* > 0.05). After the transformation (y’ = log10 (y + 1), y = original data value), the data fulfilled the abovementioned conditions. To determine differences between the mean values of localities (fixed factor), populations, and plant parts (random factors), we used nested ANOVA, a very common analysis in ecological studies (levels of random factors are similar but not identical to each other). Principal component analysis (PCA) was carried out with log-transformed data in order to determine the trends, structure, and relationships of properties and grouping of individuals. Visualizing the groups of samples with boxplots enhanced our understanding of the original (nontransformed) data and helped us to make comparisons across groups. Statgraphics Plus (version 5.0; Statistical Graphics Corporation, The Plains, VA, USA), Statistica (version 10, Stat. Soft. Inc., Tulsa, OK, USA, 2011), and Addinsoft XLSTAT software (free trial version) were used.

## 5. Conclusions

The phytochemical analysis of *G. asclepiadea* based on six representative secondary metabolites showed differences in chemical profiles among populations from different mountainous sites. Due to its absence in Vlasina populations, SWZ was the most prominently indicative of the chemical fingerprint of the plants related to the geolocation. Further research is warranted to clarify the occurrence of this compound in earlier developmental stages of Vlasina populations. The contents of other studied compounds (SWM, GP, MF, ISOOR, and ISOV) significantly differed concerning the study site. Different accumulations of analyzed secondary metabolites between above- and belowground plant parts mostly contributed to the separation of groups by PCA. This study confirms the organ-specific distribution of specific secondary metabolites, which is probably directed by different environmental pressures to above- and belowground plant parts. Moreover, it points to the indicative value of the studied compounds for the phytochemical profiling of populations from specific locations.

## Figures and Tables

**Table 1:** Results of nested ANOVA for the effects of locality (A), population (B), and plant part (C) on the content of six representative secondary metabolites in *Gentiana asclepiadea*.

Source ^1^	Sum of Squares	df	Mean Square	*F*-Ratio	*p*-Value
Swertiamarin (SWM)					
(A) locality	0.295823	1	0.295823	8.86	**0.0409**
(B) population	0.133747	4	0.0334368	0.22	0.9173
(C) part	0.909423	6	0.15157	47.18	**0.00001**
residual	0.237739	74	0.00321269		
Total (corrected)	1.58586	85			
Gentiopicrin (GP)					
(A) locality	0.0828173	1	0.0828173	2.3	0.2037
(B) population	0.144	4	0.0360001	0.12	0.9695
(C) part	1.77605	6	0.296008	19.07	**0.00001**
residual	1.14839	74	0.0155188		
Total (corrected)	3.15653	85			
Mangiferin (MGF)					
(A) locality	0.280816	1	0.280816	8.38	**0.0443**
(B) population	0.134121	4	0.0335302	0.01	0.9998
(C) part	20.8212	6	3.4702	162.47	**0.00001**
residual	1.58054	74	0.0213586		
Total (corrected)	22.8178	85			
Isoorientin (ISOOR)					
(A) locality	0.0151992	1	0.0151992	0.45	0.54
(B) population	0.135884	4	0.033971	0.06	0.9904
(C) part	3.17439	6	0.529066	44.35	**0.00001**
residual	0.882783	74	0.0119295		
Total (corrected)	4.21135	85			
Isovitexin (ISOV)					
(A) locality	0.00913338	1	0.00913338	0.14	0.7282
(B) population	0.263262	4	0.0658155	0.45	0.77
(C) part	0.877645	6	0.146274	18.3	**0.00001**
residual	0.591491	74	0.00799312		
Total (corrected)	1.73869	85			
Sweroside (SWZ)					
(A) locality	1.78663	1	1.78663	1015.34	**0.00001**
(B) population	0.00701807	4	0.00175452	0	1
(C) part	3.69257	6	0.615428	114.6	**0.00001**
residual	0.397395	74	0.0053702		
Total (corrected)	5.89691	85			

^1^ The model consists of three factors: locality, population, and part of the plant; locality is a fixed factor, population and plant part are random factors; factor population is nested within factor locality, factor plant part is nested within factor population. The bold values indicate statistically significant results (*p* < 0.05).

**Table 2:** Squared cosin es of the factor—correlations of variables.

	F1	F2	Sum
SWM	0.365	**0.472**	**0.837**
GP	**0.407**	0.388	**0.794**
MGF	**0.762**	0.123	**0.885**
ISOOR	**0.597**	0.085	0.683
ISOV	0	0.248	0.248
SWZ	**0.547**	0.138	0.685

The bold values correspond to the largest values of squared cosine between variables and factors.

**Table 3:** Geographic locations and climate characteristics of studied populations.

Mountain	Location/Population	Latitude	Longitude	Altitude	MAT ^1^ (°C)	AP ^1^ (mm)
Vlasina	Del (V1)	42.81444	22.27028	1352	5.42	720
Vlasina	Dževrljanka (V2)	42.79694	22.26222	1368	5.18	727
Vlasina	Komančićeva mahala (V3)	42.75528	22.33639	1280	5.75	705
Golija	Bele vode (G1)	43.41278	20.28361	1460	4.65	893
Golija	Golijska reka (G2)	43.36306	20.25667	1406	4.54	893
Golija	Daićko jezero (G3)	43.42444	20.26361	1455	4.89	879

^1^ Data obtained from the WorldClim set of global climate layers with a spatial resolution of 30 arc s for the period 1970–2000 [50]; MAT—mean annual temperature; AP—annual precipitation.

## Data Availability

The data presented in this study are available from the authors.
